# Long‐Pulsed Alexandrite Laser for Scrotal Angiokeratomas: Efficacy and Safety in a Clinical Study

**DOI:** 10.1111/jocd.70591

**Published:** 2025-12-08

**Authors:** Amr Molla, Mohammed Alraddadi

**Affiliations:** ^1^ Department of Medicine College of Medicine, Taibah University Madinah Saudi Arabia; ^2^ Department of Medicine College of Medicine, University of Tabuk Tabuk Saudi Arabia

**Keywords:** 755 nm, Alexandrite laser, angiokeratomas, dermatologic treatment, fordyce angiokeratoma

## Abstract

**Introduction:**

Scrotal angiokeratomas or Angiokeratoma of Fordyce (AF) are benign vascular lesions that may cause cosmetic concerns and recurrent bleeding, prompting patients to seek treatment. Various laser modalities have been explored for their management, but data on the efficacy of the long‐pulsed alexandrite laser (755 nm) in treating symptomatic cases remain limited.

**Objective:**

This study aimed to evaluate the efficacy and safety of long‐pulsed alexandrite laser for the treatment of symptomatic AF.

**Methods:**

This was a nonrandomized interventional observational study conducted at a dermatology clinic in a private hospital. Ten symptomatic male patients (*n* = 10; aged 25–67 years) with histologically confirmed scrotal angiokeratomas underwent two to three sessions of long‐pulsed alexandrite laser treatment. Clinical outcomes were assessed over a 3‐month follow‐up period, including lesion clearance, symptom resolution, patient satisfaction, and adverse effects. Statistical analysis was performed using the Wilcoxon signed‐rank test.

**Results:**

Among the 10 patients, 7 (70%) achieved measurable clearance, including 5 (50%) with near‐complete resolution and 2 (20%) with moderate improvement. Three patients (30%) showed no improvement in lesion clearance. Recurrent bleeding was initially reported by nine patients (90%); all experienced reduced frequency, and most reported complete cessation. Adverse effects occurred in five patients (50%), with some experiencing more than one event (erythema, scarring, or hypopigmentation), accounting for cumulative percentages exceeding 100%. A significant improvement in clearance was confirmed by the Wilcoxon signed‐rank test (*p* = 0.0118).

**Conclusion:**

Long‐pulsed Alexandrite laser demonstrated high efficacy and safety in treating symptomatic scrotal angiokeratomas, achieving significant lesion clearance and symptom relief with minimal adverse effects. This study supports its role as a noninvasive alternative to surgical approaches, although larger, randomized studies are recommended to validate these findings.

## 1 Introduction

Angiokeratomas are a diverse group of benign vascular lesions that present as hyperkeratotic papules with a characteristic blue‐to‐red hue and a scaly surface. Although first described in the medical literature by John Addison Fordyce in 1896 in a 60‐year‐old male [1], angiokeratomas have since been categorized into various subtypes based on clinical presentation and anatomical location. The scrotal angiokeratoma, or Angiokeratoma of Fordyce (AF), is the most common form, usually presenting as small, symptom‐free papules on the scrotum or vulva. Some lesions enlarge over time and may bleed or cause discomfort, leading patients to seek treatment [2]. Histologically, angiokeratomas are defined by the presence of dilated thin‐walled vessels confined to the superficial dermis, often accompanied by epidermal hyperplasia [3]. The prevalence of angiokeratomas in the general population has been estimated at 0.16% in earlier studies; however, recent epidemiological data are limited, and the actual prevalence may be underreported due to the asymptomatic nature and underdiagnosis of the condition [4].

Angiokeratomas occur as localized or systemic variants. Localized forms include solitary, Fordyce, circumscriptum, and Mibelli types, each with distinct features [5]. The systemic variant (angiokeratoma corporis diffusum) is linked to metabolic disorders such as Fabry disease [2, 3]. While systemic angiokeratomas often indicate an underlying lysosomal storage disease, isolated scrotal lesions have traditionally been considered a benign, age‐related phenomenon. However, emerging evidence suggests that their presence may occasionally warrant further metabolic evaluation [6]. Histological features remain consistent across types despite clinical variation [7].

The prevalence of scrotal angiokeratomas is uncertain because large epidemiologic studies are lacking and many cases go unreported [2, 8]. Although most lesions need no treatment, symptomatic ones can cause recurrent bleeding, infection, or cosmetic distress [4, 9]. AF may also mimic other dermatoses such as melanoma or genital warts, occasionally resulting in misdiagnosis [3].

AF is difficult to manage when lesions are extensive because conventional methods (excision, cryotherapy, electrocautery) often cause scarring or bleeding [4]. Laser therapy offers greater precision, less scarring, and the ability to treat multiple lesions efficiently [10]. Although several laser types have been explored—including argon, pulsed dye (PDL), neodymium‐doped yttrium aluminum garnet (Nd:YAG), copper vapor, potassium titanyl phosphate, carbon dioxide, and erbium‐doped yttrium aluminum garnet (Er:YAG) lasers—their effectiveness varies based on lesion characteristics [11]. Among these, the Long‐Pulse Alexandrite Laser (755 nm) has been extensively used for treating various types of nongenital angiokeratomas [12]. However, a recent case report demonstrated the effectiveness of long‐pulsed alexandrite laser in treating scrotal angiokeratomas [13].

This study aims to evaluate the efficacy and safety of the Long‐Pulse alexandrite Laser in the treatment of symptomatic AF. By analyzing clinical outcomes, recurrence rates, and potential complications, this research seeks to contribute valuable insights into optimizing laser‐based interventions for this condition.

## 2 Methods and Materials

### 2.1 Study Design

This was a prospective interventional observational study designed to evaluate the real‐world outcomes of long‐pulsed alexandrite laser (755 nm) for the management of symptomatic scrotal angiokeratomas. Conducted at a dermatology clinic in a private hospital in Madinah, Saudi Arabia, with a 3‐month post‐treatment follow‐up for observational assessment. Due to the low prevalence of symptomatic scrotal angiokeratomas, a feasibility‐based sample size of 10 symptomatic patients was included. Participants were enrolled using a nonprobability consecutive sampling method, ensuring the inclusion of all eligible patients who sought care at the clinic during the study period. Patients were eligible if they were male, aged 18 years or older, and had a clinically and histopathologically confirmed diagnosis of AF with symptoms such as bleeding or cosmetic concerns. Patients with systemic conditions (e.g., Fabry disease), prior treatments for angiokeratomas within the last 6 months, active infections, malignancies, or contraindications to laser therapy were excluded. This study focused on observing treatment outcomes in routine clinical practice rather than testing a novel intervention, ensuring that findings remain relevant to real‐world dermatologic care.

### 2.2 Laser Treatment Protocol and Outcome Measurements

The long‐pulsed Alexandrite laser (755 nm) was utilized with parameters tailored to lesion size. For smaller lesions, a 5 mm spot size, pulse width of 10–20 ms, and fluence of 125–175 J/cm^2^ were used. For larger lesions, a 10 mm spot size, pulse width of 5–10 ms, and fluence of 10–30 J/cm^2^ were applied. These settings were selected based on established guidelines for vascular lesions, with higher fluence applied to smaller spot sizes to ensure effective energy delivery while minimizing the risk of adverse effects [12]. Topical anesthesia with lidocaine‐prilocaine cream was applied 1 h before treatment, which consisted of two to three sessions spaced 2 weeks apart. Follow‐up visits were conducted at 4, 8, and 12 weeks post‐treatment.

Outcome measurements included clinical evaluation using baseline and follow‐up photographs to assess lesion clearance rates, recurrence within 3 months, patient‐reported satisfaction via a validated questionnaire addressing cosmetic improvement and symptom relief—rated on a 5‐point Likert scale ranging from 1 (very dissatisfied) to 5 (very satisfied)—and documentation of adverse events such as hypopigmentation and scarring. Additionally, pain was assessed during and immediately after treatment using a Numeric Rating Scale (NRS) ranging from 0 (no pain) to 10 (worst imaginable pain) to evaluate patient comfort and tolerability. This comprehensive protocol ensured a thorough evaluation of the laser's efficacy and safety.

### 2.3 Data Analysis

SPSS software (version 29.0, IBM Corp., Armonk, NY, USA) was used for all analyses. Descriptive statistics summarized patient demographics, baseline lesion characteristics, and outcomes. Two‐tailed Wilcoxon signed‐rank tests were used to compare pre‐ and postintervention continuous variables due to the small sample size. Adverse events were reported as frequencies and percentages. Because of the limited number of participants, correlation analyses were not performed; instead, results were interpreted with emphasis on clinical relevance, and effect sizes (rank biserial correlation, r) were reported where applicable to complement statistical significance. A *p*‐value < 0.05 was considered statistically significant.

### 2.4 Ethical Considerations

The study protocol was reviewed and approved by an institutional review board (IRB) in Saudi Arabia (Ref. No. UT‐547‐277‐2025) on February 27, 2025. All participants provided written informed consent for laser treatment, clinical photography, and the use of anonymized data for publication. The study was observational and noninterventional; therefore, registration as a clinical trial was not required under ICMJE, WHO, or Saudi Arabia bioethics definitions. No randomization or control group was included, as all patients received the same FDA‐approved long‐pulsed alexandrite laser, an established therapy for vascular lesions.

## 3 Results

This study included 10 male patients diagnosed with symptomatic AF, with a mean age of 42.1 years (range 25–67 years). The majority had Fitzpatrick skin types III and IV while two patients (2/10, 20%) had type V and one (1/10, 10%) had type II. Fitzpatrick skin types refer to a standardized phototype classification ranging from I (very fair skin that burns easily) to VI (very dark skin that rarely burns), based on melanin content and response to ultraviolet exposure. The duration of AF varied widely, with some cases persisting for more than two decades. Lesion sizes ranged from 1 to 15 mm, and the number of lesions per patient ranged from 12 to 40 (Table 1).

**TABLE 1:** Baseline characteristics, treatment indications, and outcomes of patients with scrotal angiokeratomas treated with long‐pulse Alexandrite laser.

Patient number	Age (Y)	Sex	Fitzpatrick skin type	Duration of AF (Y)	Size of AF	Numbers of AF	Number of sessions	Treatment indications		Results		Adverse effects	*p* ^a^
								Disfigurement	Bleeding	disfigurement improvement	Bleeding outcome		
1 (Figure 1)	40	Male	II	10	3–7 mm	35	2	No	Always	(75%) Moderate	No further bleeding	Minimal scarring with mild erythema	0.0118
2	39	Male	III	7	5–15 mm	40	2	Yes	Always	(> 90%) Near‐complete	No further bleeding	Minimal scarring with postinflammatory hypopigmentation	
3 (Figure 2)	43	Male	III	8	2–3 mm	12	2	No	Always	(> 99%) Near‐complete	No further bleeding	Mild erythema	
4 (Figure 3)	32	Male	III	10	5 to 15 mm	35	2	Yes	Always	(> 90%) Near‐complete	No further bleeding	Mild erythema	
5	45	Male	IV	9	3–7 mm	25	3	Yes	No	(50%) Moderate	N/A	Minimal scarring with postinflammatory hypopigmentation	
6 (Figure 4)	67	Male	IV	20	1–2 mm	15	2	No	Always	(> 99%) Near‐complete	No further bleeding	No adverse effects	
7	51	Male	IV	25	1–2 mm	17	3	No	Always	No improvement^b^	Less frequently bleeding	No adverse effects	
8	44	Male	IV	10	2–5 mm	25	3	No	Always	(> 90%) Near‐complete	No further bleeding	No adverse effects	
9	33	Male	V	7	2–5 mm	15	3	Yes	Frequently	No improvement	No further bleeding	No adverse effects	
10	25	Male	V	8	1–2 mm	18	3	No	Always	No improvement	Less frequently bleeding	No adverse effects	
Summary	Lesion clearance: Near‐complete (≥ 90%): five patients (50%); Moderate (50%–75%): two patients (20%); No improvement: three patients (30%). Bleeding outcome: Complete cessation in 9/9 patients (100%). Adverse effects: five patients (50%), with overlap in some cases.												

^a^ *p*‐value calculated via the Wilcoxon signed‐rank test comparing pre‐ and post‐treatment percentages.

^b^ No improvement indicates stable lesion size without reduction in disfigurement; bleeding outcomes are reported separately in the adjacent column.

Patients underwent two or three sessions of long‐pulsed alexandrite laser (755 nm) treatment, depending on lesion characteristics. The primary indication was recurrent bleeding (9/10, 90%), while cosmetic concerns motivated 4/10 (40%) patients. Post‐treatment assessments revealed a high clearance rate: 7/10 (70%) patients achieved measurable improvement, mainly among those with Fitzpatrick skin types II–IV. Among these, 5/10 (50%) achieved near‐complete clearance (≥ 90%), and 2/10 (20%) had moderate improvement (50%–75%). Three of 10 patients (30%) with skin types IV and V showed no improvement in lesion clearance. The median clearance across all patients was 78%, reflecting consistent efficacy across the cohort.

Pain levels were low, with a mean NRS pain score of 2.1 ± 0.9 (range 1–4). Four patients (4/10, 40%) reported only minimal discomfort, and no patient reported severe pain. All patients with recurrent bleeding (9/9, 100%) reported reduced frequency or complete cessation of bleeding.

**FIGURE 1:**
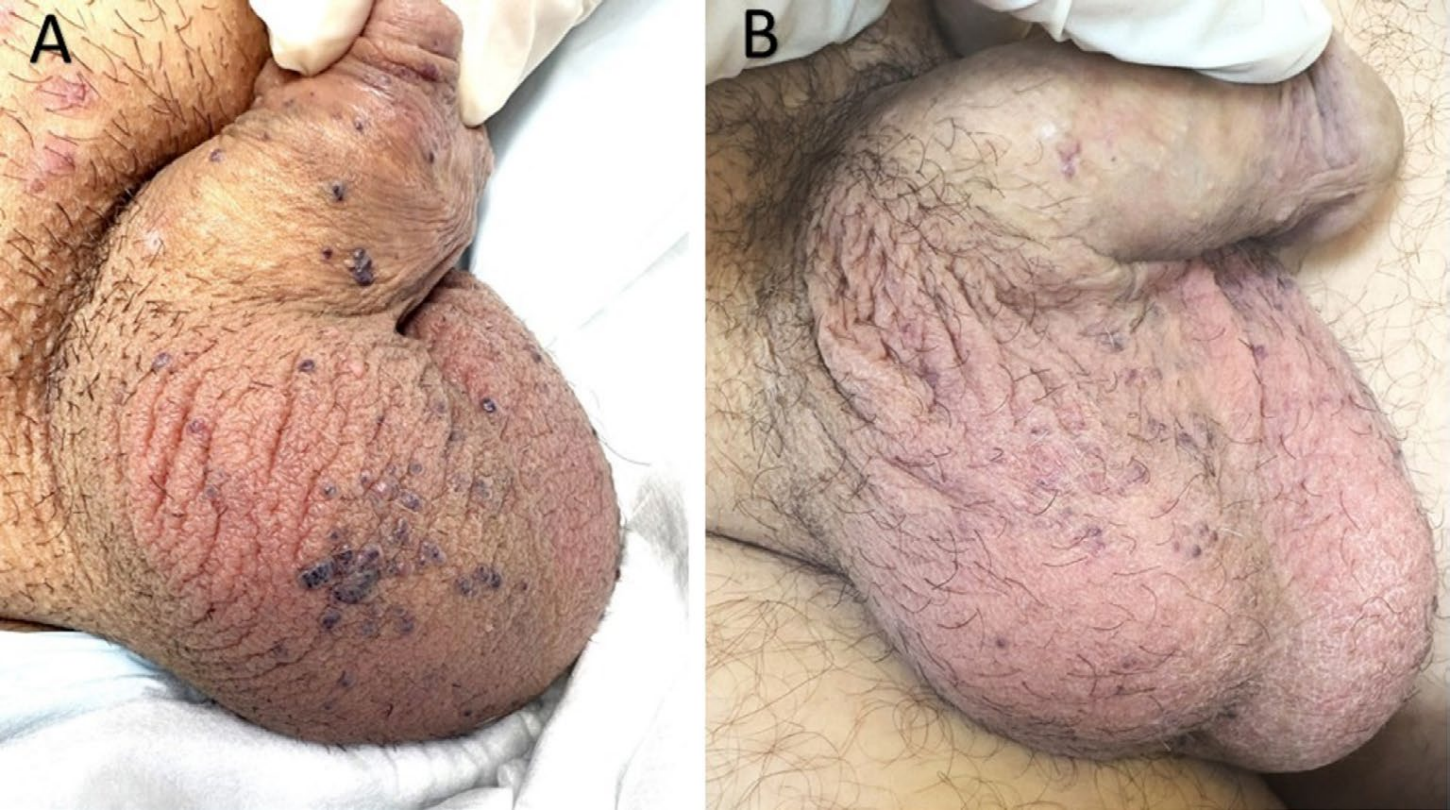
(A) A 40‐year‐old man with multiple dark‐blue papules (3–7 mm in diameter) on the scrotum and penile shaft before treatment. (B) One month after the last treatment session, a significant reduction in lesion size and number is observed.

**FIGURE 2:**
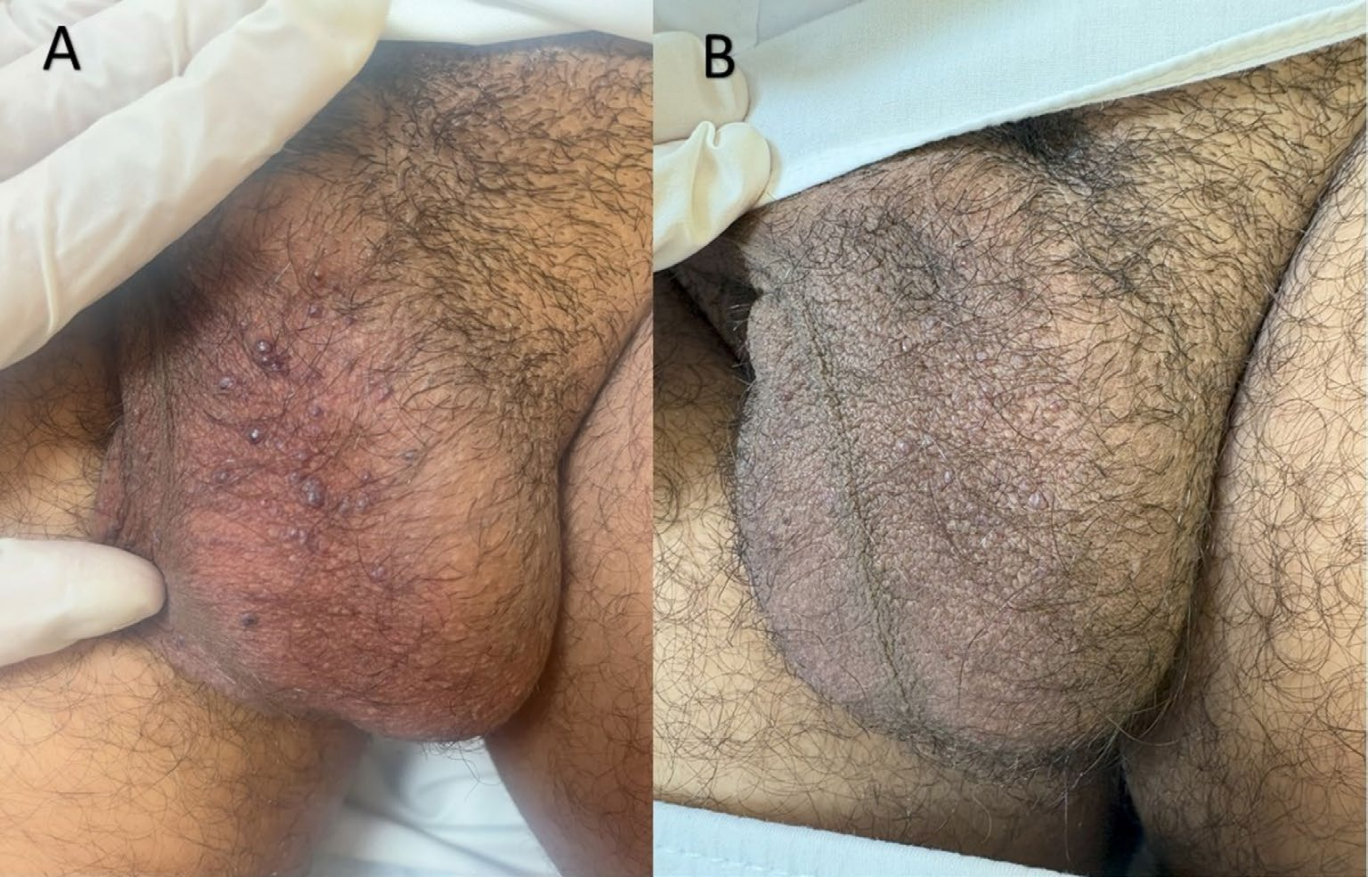
(A) A 43‐year‐old man presenting with multiple red‐to‐violaceous, velvety papules (2–3 mm in diameter) scattered over the scrotum before treatment. (B) One month after the final treatment session, complete resolution of the lesions is observed.

**FIGURE 3:**
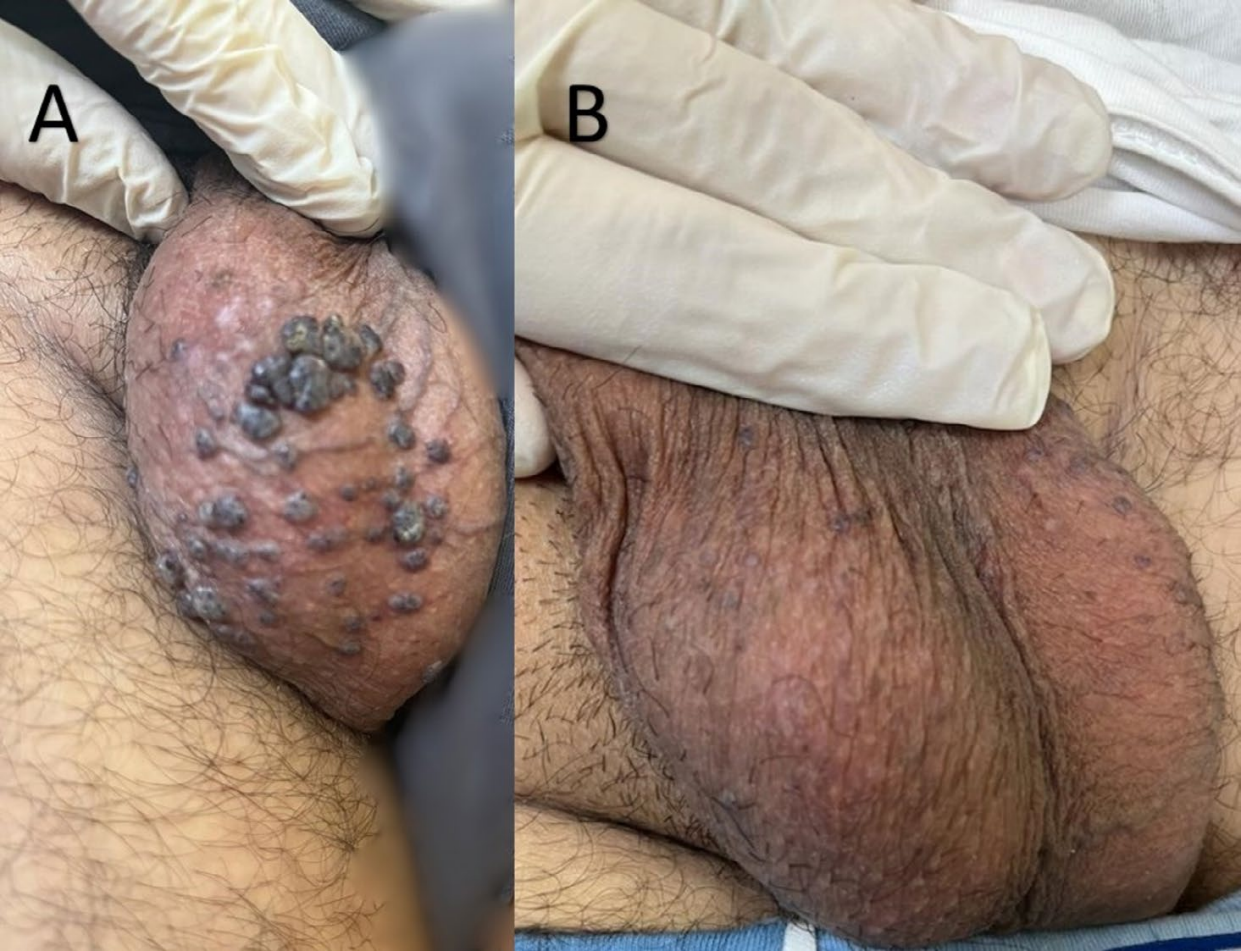
(A) A 32‐year‐old man with multiple clustered filiform hyperkeratotic red‐blue papules and nodules (5–15 mm in diameter) on the scrotum before treatment. (B) One month following the final treatment session, marked improvement is noted.

**FIGURE 4:**
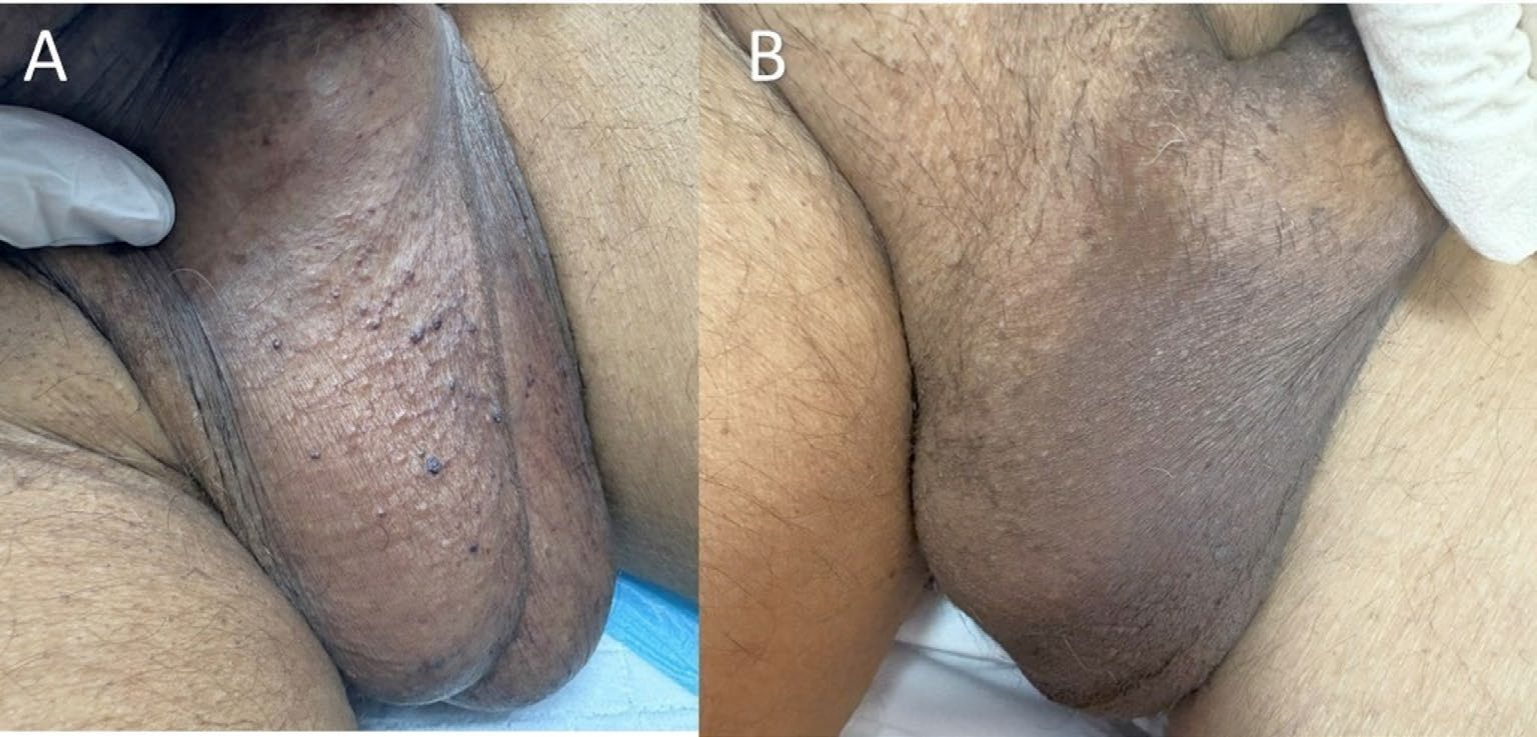
(A) A 67‐year‐old man with numerous tiny dark red papules (1–2 mm in diameter) distributed over the scrotum prior to treatment. (B) One‐month post‐treatment, substantial lesion clearance is evident.

Patient satisfaction was high, with a mean score of 4.2 out of 5 on a 5‐point Likert scale, indicating favorable perceptions of cosmetic outcomes and symptom relief. Qualitative feedback reflected enhanced self‐confidence and relief from bleeding (Figures 1, 2, 3, 4).

Statistical analysis using the Wilcoxon signed‐rank test showed a significant improvement in lesion clearance (*p* = 0.0118), supporting the efficacy of the alexandrite laser for AF treatment. Adverse effects were mild and occurred in 5/10 (50%) patients: 3/10 (30%) experienced transient erythema, 3/10 (30%) had minimal scarring, and 2/10 (20%) developed postinflammatory hypopigmentation. Because some patients reported more than one adverse effect, the cumulative percentage exceeded 100%. No major complications, such as ulceration, persistent pain, or infection, were observed (Table 1).

These findings demonstrate that long‐pulsed alexandrite laser is a highly effective and well‐tolerated treatment for AF, with strong lesion clearance, an excellent safety profile, and high patient satisfaction.

## 4 Discussion

This study aimed to evaluate the efficacy and safety of the long‐pulsed alexandrite laser (755 nm) in the treatment of AF. The findings indicate that this laser modality achieves a high clearance rate, with 70% of patients demonstrating remarkable improvement. Meanwhile, 50% of patients show more than 90% lesion resolution and complete cessation of bleeding in symptomatic cases. The treatment was well tolerated, with mild and self‐limiting adverse effects, reinforcing its potential as a promising noninvasive option for angiokeratoma management.

The observed clinical efficacy aligns with the known mechanism of action of the long‐pulsed alexandrite laser, which selectively targets vascular lesions through hemoglobin absorption, leading to vessel coagulation and subsequent lesion clearance [12]. The results of this study are consistent with previous research on laser treatments for angiokeratomas. A study utilizing the long‐pulsed Nd:YAG laser reported a comparable efficacy range (65%–100%) in a cohort of 10 patients, with only one case of long‐term atrophic scarring [14]. Given that both the Nd:YAG and alexandrite lasers operate on similar photothermolysis principles, these findings reinforce the role of laser therapy in vascular lesion management. Additionally, comparative studies on nongenital angiokeratomas treated with other laser modalities, including those referenced [15, 16], suggest that laser treatment is a highly effective alternative to surgical excision, with minimal recovery time and lower risk of complications.

In comparison to other vascular laser modalities, the long‐pulsed alexandrite laser demonstrates promising efficacy and safety for AF. A randomized, observer‐blinded trial comparing 595‐nm pulsed dye laser (PDL) and 1064‐nm long‐pulse Nd:YAG laser in angiokeratoma of Fordyce found a mean improvement of 77.6% with Nd:YAG versus 61.8% with PDL (*p* < 0.005) [17]. Recurrence rates in Nd:YAG studies are generally low but not absent, with occasional late scarring reported [14]. PDL, while effective, has been associated with higher recurrence and pigmentary alteration in darker skin types [10]. Surgical excision, historically used for symptomatic or extensive cases, offers definitive clearance but carries greater risks of scarring, hemorrhage, and longer recovery [18]. Although our study did not include direct comparisons, the alexandrite laser operates at an intermediate wavelength (755 nm), balancing hemoglobin absorption and melanin safety. This potentially offers deeper penetration than PDL and a lower risk of post‐treatment pigmentary change or scarring compared to Nd:YAG, particularly in lighter skin types. Moreover, alexandrite systems are often more cost‐effective than Nd:YAG platforms, offering comparable treatment duration, high patient satisfaction, and minimal downtime. Further head‐to‐head trials are warranted to delineate these advantages in terms of efficacy, recurrence prevention, safety, and economic feasibility.

The observed variation in treatment response based on Fitzpatrick skin types may be attributed to the differential absorption and scattering of laser energy in varying melanin densities. In lighter skin types (II and III), melanin minimally competes with hemoglobin for laser absorption, allowing deeper and more selective vascular targeting by the alexandrite laser (755 nm). Conversely, in darker skin types (V), increased epidermal melanin absorbs more of the laser energy superficially, reducing effective penetration to dermal vessels and increasing the risk of epidermal injury. This photothermal competition may explain the reduced efficacy and greater treatment resistance observed in patients with darker skin tones [19].

A major strength of this study is that it represents the first investigation to systematically evaluate the safety and efficacy of Long‐Pulsed alexandrite Laser for the treatment of AF in a larger cohort within a real‐world clinical setting, rather than a single case report. Unlike prior anecdotal reports, this study provides a more comprehensive and generalizable assessment by utilizing objective clinical parameters, standardized treatment protocols, and consistent follow‐up measures, thereby enhancing the reliability and clinical relevance of the findings. However, this study is limited by its small sample size (*n* = 10) and the exclusive focus on scrotal lesions, which may affect the statistical power and generalizability of the findings. Nevertheless, symptomatic scrotal angiokeratomas are rare, and most published laser studies on this condition report similarly small cohorts. This study is limited by its observational design and the absence of a control group, which restricts direct comparisons with other treatment modalities. Additionally, the short 3‐month follow‐up period may not adequately capture late recurrences or delayed adverse effects. While selection bias and the subjective assessment of outcomes are potential limitations, these were mitigated by using standardized laser protocols, prospective follow‐up, and clinical documentation through serial photography. These factors support the study's real‐world relevance and add meaningful evidence to the limited literature on this uncommon condition.

Further studies with larger cohorts, diverse anatomical locations, and long‐term follow‐up are needed to validate the efficacy and safety profile across broader patient populations. Additionally, future research should explore optimal laser parameters, combination treatments, and the potential role of adjunctive therapies to enhance clinical outcomes. Larger randomized controlled trials comparing the alexandrite laser with other treatment modalities, such as the Nd:YAG laser and pulsed dye laser, would provide more robust evidence for clinical decision‐making.

## 5 Conclusion

The findings of this study establish the long‐pulsed alexandrite laser as a highly effective, well‐tolerated, and minimally invasive treatment for AF. The high clearance rates, complete resolution of bleeding, and minimal adverse effects highlight its potential as a superior alternative to traditional surgical excision, offering precision, enhanced safety, and improved patient comfort while preserving tissue integrity. This study, being the first of its kind in a larger patient cohort, provides strong clinical evidence supporting the integration of alexandrite laser into routine dermatologic practice for AF management. However, while these results are promising, larger‐scale, multicenter studies are necessary to further validate its efficacy, optimize treatment parameters, and assess long‐term outcomes. Future research should include comparative trials with alternative laser technologies and multimodal treatment strategies to refine clinical protocols. Ultimately, this study advances the role of laser‐based therapies in vascular dermatology, reinforcing their value in safe and effective treatment paradigms.

## Author Contributions

Both authors made a significant contribution to the work reported, whether that is in the conception, study design, execution, acquisition of data, analysis and interpretation, or in all these areas; took part in drafting, revising or critically reviewing the article; gave final approval of the version to be published; have agreed on the journal to which the article has been submitted; and agree to be accountable for all aspects of the work.

## Funding

The authors have nothing to report.

## Ethics Statement

This original article is unpublished and not under consideration elsewhere. All content, except cited references, is based on our research. We adhered to Saudi ethical standards, securing IRB approval from the University of Tabuk (UT‐547‐277‐2025) on February 27, 2025, with informed consent for procedures, photography, and publication. Clinical trial registration is not required, as the study does not meet ICMJE, WHO, or Saudi Arabia Bioethics Guidelines for clinical trial classification. The study ensures confidentiality, follows the Declaration of Helsinki, and all authors have contributed significantly and approve the submission.

## Conflicts of Interest

The authors declare no conflicts of interest.

## Data Availability

The data that support the findings of this study are available on request from the corresponding author. The data are not publicly available due to privacy or ethical restrictions.
